# Post-Transcriptional Controls by Ribonucleoprotein Complexes in the Acquisition of Drug Resistance

**DOI:** 10.3390/ijms140817204

**Published:** 2013-08-20

**Authors:** Hoin Kang, Chongtae Kim, Heejin Lee, Wook Kim, Eun Kyung Lee

**Affiliations:** 1 Department of Biochemistry, College of Medicine, Catholic University of Korea, Seoul 137-701, Korea; E-Mails: kanghoin1@naver.com (H.K.); leostar81@naver.com (C.K.); polaliswish@naver.com (H.L.); 2 Department of Molecular Science and Technology, Ajou University, Suwon 443-749, Korea

**Keywords:** ribonucleoprotein complex, RNA binding protein, microRNAs, drug resistance, mRNA stability, translation

## Abstract

Acquisition of drug resistance leads to failure of anti-cancer treatments and therapies. Although several successive chemotherapies are available, along with efforts towards clinical applications of new anti-cancer drugs, it is generally realized that there is a long way to go to treat cancers. Resistance to anti-cancer drugs results from various factors, including genetic as well as epigenetic differences in tumors. Determining the molecular and cellular mechanisms responsible for the acquisition of drug resistance may be a helpful approach for the development of new therapeutic strategies to overcome treatment failure. Several studies have shown that the acquisition of drug resistance is tightly regulated by post-transcriptional regulators such as RNA binding proteins (RBPs) and microRNAs (miRNAs), which change the stability and translation of mRNAs encoding factors involved in cell survival, proliferation, epithelial-mesenchymal transition, and drug metabolism. Here, we review our current understanding of ribonucleoprotein complexes, including RBPs and miRNAs, which play critical roles in the acquisition of drug resistance and have potential clinical implications for cancer.

## 1. Introduction

Chemotherapy, using anti-cancer drugs, has been widely used in treatment of malignancies, however, primary or acquired drug resistance cause therapeutic failure in cancer treatment. Drug resistance is considered as a multifactorial phenomenon and results from a variety of factors including individual variations in patients and genetic and/or epigenetic differences in tumors. Several research groups have reported the mechanisms of drug resistance, including altered expression or mutation of transporter proteins that increase drug efflux from cancer cells, reduced uptake of drugs, increased repair of DNA damage and decreased sensitivity due to induction of apoptosis, and acceleration of drug metabolism [1–4]. These mechanisms play important roles in the acquisition of drug resistance. It has been known that causes of drug resistance are primarily linked to random mutational events induced by anti-cancer drugs [5–8]. Recently, a number of studies have indicated that non-mutational regulation of gene expression, including microRNAs (miRNAs), is also largely involved in the acquisition of drug resistance [9,10]. However, the molecular and cellular mechanisms of drug resistance acquisition have not yet been fully elucidated.

Gene expression is tightly regulated at both the DNA and protein level in response to various extracellular stimuli. Accumulating evidence indicates that there are fine-tuning steps in the regulation of gene expression at the mRNA level [11]. This is executed by post-transcriptional regulators, such as RNA binding proteins (RBPs) and miRNAs. In eukaryotic cells, all RNAs are associated with RBPs co-transcriptionally and form ribonucleoprotein (RNP) complexes, which are composed of, not only various proteins, but also RNAs such as small nuclear RNAs (snRNAs) and miRNAs [12–14]. RBPs are main components of RNP complexes and affect a variety of important biological functions in eukaryotic organisms, including cell proliferation, apoptosis, signal transduction, and drug resistance by regulating mRNA splicing, stability, storage, and translational efficiency [15]. RBPs dynamically regulate the fate of mRNAs by interacting with mRNAs through their RNA binding domains, such as RNA recognition motif (RRM), K-homology (KH) domain, RGG (Arg-Gly-Gly) box, DEAD/DEAH box, zinc finger, double stranded RNA-binding domain (dsRBD), and Piwi/Argonaute/Zwille (PAZ) domain [14]. Differential expression or defects of RBPs are related to several human diseases such as neuropathies, muscular atrophies and cancers [16,17]. Another post-transcriptional regulator miRNAs are small noncoding RNAs involved in the regulation of gene expression. They mainly bind to the 3′ untranslated region (UTR) of target mRNAs by forming incomplete base-pairing with target mRNAs, leading to mRNA destabilization or repression of translation by loading to RNA-induced silencing complexes (RISCs) [18]. Increasing evidence indicates that the acquisition of drug resistance is mediated by post-transcriptional events. Post-transcriptional regulators exert various regulatory functions in different cells and tissues, or in response to different types of anti-cancer drugs. Here, we will review recent findings on post-transcriptional regulation of drug resistance-related genes by RNP complexes, including RBPs and miRNAs.

## 2. RBPs and Drug Resistance

In eukaryotic cells, RBPs and small noncoding RNAs, such as miRNAs, constitute the RNP with mRNAs and individual RNP components could function as adaptors that allow mRNAs to interact with several factors regulating their stability, translation, splicing, and subcellular localization [12,19]. RBPs that regulate turnover and translation (sometimes named TTR-RBPs) interact with the specific regions of target mRNAs via various RNA binding domains [14,20]. The majority of RBPs have been shown to regulate mRNA metabolism by associating with the 3′UTR of target mRNAs, and in some cases, with not only 5′UTR, but also coding region, thereby regulating various cellular function such as cell proliferation, tumorigenesis, cell death, differentiation [11,20]. In this section, we will discuss recent findings showing the regulation of drug resistance by several RBPs.

### 2.1. HuR

#### 2.1.1. Regulation of mRNA Stability and Drug Resistance

RBPs can affect mRNA turnover by regulating mRNA stability. Several studies have shown a molecular link between regulation of mRNA stability by RBPs and drug resistance. HuR, a member of the human antigen (Hu) family, is an RNA-binding protein regulating splicing, mRNA stability, and translational rates of target mRNAs [21–23]. It is known that HuR is involved in acquisition of drug resistance by regulation of mRNA stability. Hostetter *et al.* demonstrated the regulatory mechanism of tamoxifen resistance mediated by HuR [24]. Tamoxifen blocks estrogen signaling through the estrogen receptor (ER) by competing with estrogen, and prolonged exposure of tamoxifen leads to development of resistance. Tight regulation of ER expression and downstream signaling cascades are responsible for the acquisition of tamoxifen resistance. The interaction between HuR and *ER* mRNA, through its 3′UTR, was shown by Pryzbylkowski and colleagues [25]. This binding was lost after treatment with DNA methyltransferase inhibitors or histone deacetylase inhibitors, leading to *ER* mRNA destabilization. Furthermore, tamoxifen increased cytoplasmic localization of HuR, resulting in tamoxifen resistance in MCF cells by stabilizing *ER* mRNA [25]. Consistent with this result, downregulation of cytoplasmic HuR using a JNK inhibitor reduced *ER* mRNA stability and increased tamoxifen responsiveness, whereas overexpression of HuR resulted in tamoxifen resistance in MCF cells by stabilizing *ER* mRNA.

Hsia *et al.* presented lapatinib-mediated upregulation of aggressiveness in breast cancer cells [26]. Lapatinib, a dual epidermal growth factor receptor (EGFR) and human epidermal growth factor receptor 2 (HER2) kinase inhibitor, is used for the treatment of advanced HER2-positive breast cancer [27]. It was shown that upregulated EGFR expression by lapatinib treatment facilitated the association between EGFR and HuR, resulting in *cox-2* mRNA stabilization. *Cox-2* mRNA stabilization is responsible for the enhanced aggressiveness of breast cancer cells by increasing cell migration as well as invasion.

In hepatocarcinoma cells, arsenic trioxide (ATO), a chemotherapeutic agent treatment, enhanced the binding of HuR to *TG-interacting factor* (*TGIF*) mRNA and upregulated TGIF expression [28]. Downregulation of HuR inhibited TGIF expression by destabilizing *TGIF* mRNA and, this, enhanced ATO-induced cell death in HepG2 cells.

#### 2.1.2. Regulation of Translation and Drug Resistance

The important role of HuR in translational regulation is continually elucidated by numerous studies. In this section, a molecular link between HuR-controlled translation and drug resistance will be discussed. Constantino *et al*. implicated HuR in the regulation of gemcitabine efficacy in pancreatic cancer [29]. Deoxycytidine kinase (DCK) is an enzyme, which metabolizes several anti-cancer chemotherapeutic agents including gemcitabine [30]. The levels of DCK correlated with overall patient survival after gemcitabine-based therapy in pancreatic ductal adenocarcinoma (PDA) specimens [31]. Overexpression of HuR increased sensitivity to gemcitabine by facilitating *DCK* mRNA translation in pancreatic cancer cells [29]. Accumulated cytoplasmic HuR, caused by gemcitabine treatment, resulted in upregulation of DCK expression by enhancing the association between HuR and *DCK* mRNA. Interestingly, it was shown that PDA patients with low cytoplasmic HuR levels had an increased risk of mortality compared with patients with high HuR levels [29]. In glioma cells, HuR regulates B-cell lymphoma 2 (BCL2) expression through its 3′UTR during chemotherapeutic agent-induced apoptosis [32]. HuR silencing reduced glioma cell proliferation, accompanied by concomitant induction of apoptosis and reduction in tumor volume. In contrast, HuR overexpression resulted in chemoresistance to standard glioma therapeutic agents such as etoposide, topotecan, and cisplatin by stabilizing *BCL2* mRNA as well as enhancing translational efficiency. In ovarian cancer cells, HuR enhanced tubulin beta III (TUBB3) in conjunction with miR-200c [33,34]. Nuclear HuR allowed inhibitory action of miR-200c on *TUBB3* mRNA and, conversely, cytoplasmic HuR enhanced translation of TUBB3, which is implicated in a poor outcome.

Several studies reported HuR function during the cellular response to doxorubicin, an anti-cancer drug [35,36]. Latorre *et al*. showed that HuR was significantly downregulated in doxorubicin-resistant MCF7 cells that overexpress the multidrug resistance (MDR)-related ABCG2 transporter, and the results were consistent with downregulation of HuR target genes, as well as loss of rottlerin toxicity. HuR was also involved in the regulation of TOP2A translation and doxorubicin efficacy in HeLa cells [36]. Srikantan *et al*. showed that competitive binding between HuR and miR-548c-3p to *TOP2* mRNA resulted in differential effectiveness of doxorubicin. Downregulation of TOP2A due to HuR silencing or miR-548c-3p expression selectively decreased DNA damage after doxorubicin treatment and, consequently, increased drug resistance.

### 2.2. RBM

RNA binding motif protein 3 (RBM3) is a glycine-rich protein containing an RRM and interacts with several RNAs [37]. In breast and ovarian cancers, it was shown that RBM3 expression levels in cisplatin-sensitive cells were significantly higher than in resistant cells [38,39]. RBM3 silencing resulted in increased resistance to cisplatin in A2780 cells [39]. However, the function of RBM3 during regulation of cisplatin resistance was not explored. Another nuclear RBP RBM5 (also known as g15, LUCA-15, and H37) was also involved in the regulation of cisplatin resistance [40]. Li *et al*. reported lower expression of RBM5 in cisplatin-resistant non-small cell lung cancer A549/DDP cells. Overexpression of RBM5 in resistant A549/DDP cells restored the response to cisplatin; in contrast, RBM5 silencing in A549 cells inhibited cisplatin-induced apoptosis. Although RBM modulated cell growth and apoptosis in response to cisplatin, the role of RBM5 in the acquisition of drug resistance needs to be further elucidated.

### 2.3. IMP3

IMP3, a member of the insulin-like growth factor II mRNA binding proteins (IMPs), is involved in the regulation of drug resistance in breast cancer [41]. Downregulation of IMP3 in breast cancer cells led to increased sensitivity to doxorubicin and mitoxantrone by regulating the expression of breast-cancer resistance protein (BCRP). IMP3 association with *BCRP* mRNA, and downregulation of IMP3 by lentivirus, lowered the levels of both *BCRP* mRNA and BCRP protein. Restoration of BCRP expression in IMP3-downregulated cells increased chemoresistance.

### 2.4. CUG Binding Protein 1

CUG binding protein 1 (CUG-BP1) is also involved in the acquisition of drug resistance. Augmented expression of CUG-BP1 in oesophageal cancers was responsible for the upregulation of survivin, a member of the inhibitors of apoptosis protein (IAP) family, by stabilizing *survivin* mRNA [42]. Overexpression of CUG-BP1 in oesophageal epithelial cells resulted in enhanced survivin expression and consequent increased resistance to apoptosis, whereas CUG-BP1 siRNA transfection made cells more susceptible to chemotherapy.

### 2.5. Butyrate Response Factor 1

Butyrate response factor 1 (BRF1) is one of the AU-rich element (ARE) binding proteins and plays a role in the degradation of target mRNAs [43]. Lee *et al*. identified *BRF1* as one of the genes upregulated in cisplatin-sensitive head and neck squamous cell carcinoma (HNSCC)-derived cells [44]. It was shown that BRF1 downregulated the expression of cIAP, a member of the IAP family, by destabilizing *cIAP* mRNA. BRF1 overexpression increased cisplatin sensitivity, whereas inhibition of BRF1 decreased cisplatin-induced apoptosis in HNSCC cells.

### 2.6. Other RBPs Implicated in Drug Resistance

Stark *et al*. reported the role of heterogeneous ribonucleoprotein H1/H2 (hnRNP H1/H2) in splicing of pre-thymidine phosphorylase (*TP*) mRNA [45]. It was shown that downregulation of TP in drug-resistant cancer cells resulted from abnormal splicing of its precursor mRNA. hnRNP H1/H2 mediated aberrant *TP* mRNA splicing, therefore, resulting in the acquisition of drug resistance to TP-activated fluorepyrimidine anti-cancer drugs. Augmented expression of the oncogene metadherin (MTDH, also known as AGE-1 and LYRIC) is related to metastasis and chemoresistance [46–48]. Meng *et al*. provided experimental evidence for MTDH as an RBP [49]. Downregulation of MTDH enhanced stress granule formation and reduced survival in response to chemotherapy by interacting with several cytoplasmic proteins as well as mRNAs, such as *PDCD10* and *KDM6A*.

## 3. MiRNAs and Drug Resistance

A number of studies reviewed in the above section showed that drug resistance could be established by post-transcriptional regulation, including mRNA stability, translation, subcellular localization, and maturation through splicing. In addition to RBPs, other post-transcriptional regulators, such as miRNAs, also regulate the acquisition of drug resistance in response to various anti-cancer agents. A number of studies showed relationships between drug resistance and miRNAs. The expression of miRNAs is dynamically regulated in response to various anti-cancer drugs, and could affect drug functions, such as efficacy and toxicity, by targeting many genes affecting pharmacokinetics or pharmacodynamics of drugs [50–52]. Drug-responsive miRNAs dynamically regulated during the acquisition of drug resistance are intriguing and potentially useful for the development of biomarkers for diagnosis and prognosis of cancer. In this section, we will discuss current experimental findings showing molecular evidence of miRNAs involved in the acquisition of drug resistance.

### 3.1. Cisplatin Resistance and miRNAs

Cisplatin has been used for the treatment of various types of cancers and may be considered to represent a class of platinum-containing anti-cancer drugs [53,54]. Several recent reports have implicated miRNAs in the acquisition of cisplatin resistance. In ovarian cancer cells, miR-93 was identified as an upregulated miRNAs in response to cisplatin. Overexpression of miR-93 in OVCAR3 and SKOV3 cells decreased PTEN expression, a tumor suppressor responsible for the dephosphorylation of AKT1, and resulted in an increased resistance to cisplatin [55]. In lung cancer, cisplatin-sensitive patients showed low plasma levels of miR-155, whereas miR-155 was highly expressed in patients with cisplatin resistant malignancies. miR-155 downregulated chemosensitivity of A549 cells to cisplatin by targeting Apaf-1, the apoptotic protease activating factor-1. Consistently, downregulation of miR-155 resulted in enhanced sensitivity to cisplatin through the induction of DNA damage and apoptosis via restoration of the mitochondrial apoptotic pathway [56]. miR-155 also affects cisplatin resistance in HT29 cells. Adrenaline-induced miR-155 was responsible for downregulation of phosphatase 2A catalytic subunit alpha (PPP2CA), a negative regulator of cell growth, higher cell proliferation rate, and increased resistance [57]. Zhou *et al.* reported an inverse correlation between MCL1, an anti-apoptotic protein, and miR-135a/b in A549/CDDP cells, a cisplatin-resistant human lung cancer cell line. The ectopic expression of miR-135a/b increased sensitivity by downregulating MCL1 expression and increasing cisplatin-induced apoptosis [58]. Zhu *et al*. showed that miR-181b, downregulated in the vincristine (VCR)-resistant gastric cancer cell line SGC7901/VCR and the cisplatin (CDDP)-resistant lung cancer cell line A549/CDDP, modulated multi-drug resistance by targeting BCL2 [59]. They demonstrated that overexpression of miR-181b repressed BCL2 expression as well as increased anti-cancer drug-induced apoptosis in both cell lines, indicating that miR-181b plays a role in the development of multi-drug resistance in both gastric and lung cancer cell-lines by regulating cell death via downregulation of BCL2.

In melanoma, downregulation of miR-200c was also responsible for drug resistance to cisplatin [60]. Overexpression of miR-200c led to downregulation of cell proliferation and migration capacity, which was responsible for lower expression of Bmi-1 by miR-200c. Although an association between *Bmi-1* mRNA and miR-200c has not elucidated, the effects of miR-200c on melanoma cell proliferation, migration, and resistance to cisplatin were rescued by overexpression of Bmi-1. In breast cancer, miR-203 is one of the augmented miRNAs, and inhibition of miR-203 in MCF7 cells increased drug sensitivity to cisplatin as well as apoptosis through the activation of caspase-9 and caspase-7 as well as cleavage of poly (ADP-ribose) polymerase (PARP) [61]. It was shown that the suppressor of cytokine signaling 3 (SOCS3) was the direct target of miR-203 and mediated anti-miR-203-induced cell death.

### 3.2. 5-Fluouracil (5-FU) Resistance and miRNAs

5-FU, a classical anti-metabolite, inhibits thymidylate synthase (TYMS) and is widely used for anti-cancer therapy of colon and pancreatic cancers [62]. 5-FU-related miRNAs were identified extensively in various cell types and tumors. Kurokawa *et al*. identified differentially expressed miRNAs from two types of 5-FU-resistant colon cancer cells derived from DLD-1 and KM12C cell lines [63]. In 5-FU-resistant cells, they found the upregulation of miR-19b and enhanced association of miR-19b with *SFPQ* and *MYBL2* mRNAs, which are involved in cell-cycle regulation. This was linked to 5-FU resistance in colon cancer cells. In another study, miR-192 and miR-215 were identified as post-transcriptional regulators of TYMS, a predictive biomarker for 5-FU response in colorectal cancer [64]. There was no significant correlation between *TYMS* mRNA and protein levels in colorectal cancer, and it was suggested the post-transcriptional regulation by miRNAs could be responsible for the regulation of TYMS expression. Overexpression of miR-192 and miR-215 resulted in decreased cell proliferation by targeting cell-cycle progression; however, it did not affect sensitivity to 5-FU treatment in several colorectal cell lines. In hepatocellular carcinoma, miR-195 was identified by comparison between parental BEL-7402 and 5-FU-resistant BEL-7402/5-FU [65]. Ectopic expression of miR-195 in BEL-740/5-FU cells enhanced chemosensitivity to 5-FU, whereas inhibition of miR-195 increased resistance to 5-FU by targeting Bclw, an anti-apoptotic protein. miR-195 also downregulated expression of the Bcl-2 family, leading to increased apoptosis and thereby improving sensitivity to 5-FU.

Increased expression of miR-21 is associated with poor prognosis from 5-FU chemotherapy in stage II and III colorectal cancer (CRC) [66]. Valeri *et al*. showed that the mismatch repair (MMR) core proteins, human mutS homolog 2 (hMSH2) and hMSH6, are regulated by miR-21 in CRC cells [67]. There was an inverse correlation between miR-21 and hMSH2/6 in CRC. Overexpression of miR-21 resulted in the decrease of G2/M arrest and apoptosis in SW620 and Colo320DM cells after 5-FU treatment. Additionally, in CRC xenograft models, overexpression of miR-21 induced 5-FU resistance through downregulation of hMSH2-hMSH6. In addition, Chai *et al.* showed differential expression levels of miR-20a between the colorectal cancer cells SW480 and SW620, and that elevated miR-20a in SW620 cells was responsible for drug resistance through targeting of BNIP [68].

### 3.3. Paclitaxel Resistance and miRNAs

Paclitaxel binds microtubules, thereby stabilizing them and inhibiting mitosis, and is used for treatment of several cancers. miRNA profiling was used to identify differentially expressed miRNAs between human ovarian carcinoma SKOV3 cells and paclitaxel-resistant SKOV3-TR30 cells [69]. The miR-17-92 cluster was upregulated in paclitaxel-resistant cells and inhibition of miR-17-92 led to cell cycle arrest at the G2/M phase and growth inhibition by targeting BIM and PTEN. miR-17-92 downregulated BIM expression and, thus, induced paclitaxel resistance in SKOV3-TR30 cells. In another study, miR-31 was downregulated in paclitaxel-resistant KFr13Tx cells and restoration of miR-31 resulted in enhanced sensitivity to paclitaxel [70]. miR-31 downregulated MET expression and there was an inverse correlation between miR-31 and MET in ovarian cancer cells. In non-small cell lung carcinoma A549, it was shown that miR-34c-5p was involved in the regulation of paclitaxel-induced apoptosis [71]. miR-34c-5p downregulated BCL2-modifying factor (BMF), as well as c-myc, and increased resistance to paclitaxel-induced cell death.

### 3.4. Tamoxifen Resistance and miRNAs

Tamoxifen is one of the most successful anti-cancer drugs for estrogen receptor (ER)-positive breast cancer cells. However, endocrine resistance to tamoxifen often develops. The mechanisms that underlie resistance are not fully elucidated [72,73]. It was reported that 14-3-3ζ expression was upregulated in response to tamoxifen, which correlated with an early time to disease recurrence and upregulation of 14-3-3ζ resulted from the rapid decrease of miR-451 [74,75]. The levels of miR-451 and 14-3-3ζ were inversely correlated in tamoxifen-resistant breast cancer cells. Overexpression of miR-451 suppressed cell proliferation, colony formation, and activation of HER2, EFGR, and MAPK signaling pathways. Moreover, miR-451 expression restored the growth inhibitory effectiveness of selective ER modulators (SERMs) in endocrine-resistant cells, which was responsible for the development of endocrine resistance to tamoxifen. Another study showed that miR-221/222 was upregulated in tamoxifen-resistant breast cancer cells and linked to the acquisition of tamoxifen resistance [76]. It was shown that p27, a target for miR-221/222, was reduced in tamoxifen-resistant cells as well as miR-221/222 overexpressed cells, and that ectopic expression of p27 in tamoxifen-resistant cells increased tamoxifen-induced cell death.

### 3.5. Multi-Drug Resistance and miRNAs

miR-21, a miRNA increased in various cancers, was responsible for induction of drug resistance to arestin trioxide (ATO) in acute promyelocytic leukemia (APL) [77]. It was shown that HL60 and K562 cells, overexpressing miR-21, became resistant to ATO by targeting PDCD4, a tumor suppressor involved in the regulation of cell growth, apoptosis, invasion, and cell cycle. Anti-miR-21 transfection increased G1 and sub-G1 phase-arrested cells and increased sensitivity to ATO. Robin *et al.* reported a negative correlation between EYA3, a DNA repair and transcriptional cofactor protein, and miR-708 in Ewing sarcoma samples [78]. They showed that negative regulation of miR-708 expression by EWS/FLI1 was responsible for the high expression of EYA3 as well as the resistance to etoposide and doxorubicin. Asuthkar *et al*. showed that epigenetic regulation of miR-211 by MMP-9 was responsible for the acquisition of chemoresistance to tomozolomide [79]. It was shown that miR-211 expression was decreased in grade IV glioblastoma multiforme (GBM) and related to the inhibition of invasion and migration of glioma cells through downregulation of MMP-9, and enhancement of apoptosis after temozolomide treatment. miR-215 was related to resistance to methotrexate (MTX, DHFR inhibitor) and Tomudex (TDX, TYMS inhibitor) by targeting dihydrofolate reductase (DHFR) and thymidylate synthase (TS) [80]. It was shown that ectopic expression of miR-215 induced cell cycle arrest at G2 phase and reduced cell proliferation with a p53-dependent increase in p21 expression in osteosarcoma and colorectal cancer cell lines. Recently, it was reported that lower expression of miR-143 in CRC was correlated with clinical stages and lymph node metastasis and was responsible for drug resistance by targeting the insulin-like growth factor-I receptor (IGF-IR) [81]. Overexpression of miR-143 in SW1116 cells suppressed proliferation, migration, angiogenesis, and tumorigenesis as well as decreased resistance to oxaliplatin in an IGF-IR-dependent manner.

## 4. Conclusions

In this review, we attempted to summarize the role of RBPs and miRNAs in the acquisition of drug resistance. Although numerous studies have shown that post-transcriptional regulators, especially miRNAs, were differentially expressed and involved in the direct or indirect regulation of drug resistance, there is still a considerable lack of understanding of the detailed mechanisms and intracellular pathways regulated by RBPs and/or miRNAs. We discussed recent studies showing that ribonucleoprotein complexes, including RBPs and miRNAs, could play critical roles in the regulation of anti-cancer drug efficacy by regulating their target mRNA stability and translational efficiency. Post-transcriptional regulators involved in the acquisition of anti-cancer drugs resistance were summarized in Tables 1 and 2.

Despite the development of new anti-cancer agents, acquisition of resistance acts as an obstacle to successful treatment of cancers [82]. Mechanism-based studies of drug resistance may be useful in designing strategies for the development of new therapies that are less susceptible to known resistance mechanisms. Understanding the detailed mechanism underlying the post-transcriptional regulation during the acquisition of drug resistance is an important next step for investigation, as regulatory RBPs and miRNAs represent promising new therapeutic intervention in drug resistance.

## Figures and Tables

**Table 1:** RNA binding proteins involved in the acquisition of drug resistance.

Regulators	Target genes	Anti-cancer drugs	Cell or tissue types	Function	Effect	References
HuR	ER	Tamoxifen	Breast cancer cell	mRNA stability ↑	Resistance ↑	[13]
HuR	COX-2	Lapatinib	Breast cancer cell	mRNA stability ↑	Invasion ↑ Metastasis ↑	[19]
HuR	TGIF	ATO	Hepatocellular carcinoma cell	mRNA stability ↑	Cell death ↓	[16]
HuR	dCK	Gemcitabine	Pancreatic cancer	Translation ↑	Sensitivity ↑	[17]
HuR	BCL2	Etoposide, Topotecan, Cisplatin	Glioma cell	Translation ↑	Resistance ↑	[21]
HuR	TOP2A	Doxorubicin	Hela cell	Translation ↑	Sensitivity ↑	[25]
HuR	Several targets	Doxorubicin	Breast cancer cell	Translation ↑	Sensitivity ↑	[24]
HuR	Beta-tubulin	Cisplatin	Ovarian cancer cells	Translation ↑	Resistance ↑	[22]
RBM3	Unknown	Cisplatin	Epithelial ovarian cancer	Unknown	Sensitivity ↑	[28]
RBM5	Unknown	Cisplatin	Non-small cell lung cancer	Unknown	Sensitivity ↑	[29]
IMP3	BCRP	Doxorubicin, Mtoxantrone	Breast cancer cell	mRNA stability ↑	Resistance ↑	[30]
CUG-BP1	Survivin	Camptothecin	Oesophageal cancer	mRNA stability ↑	Resistance ↑	[31]
BRF1	cIAP2	Cisplatin	Head and neck squamous Carcinoma cell lines	mRNA degradation	Sensitivity ↑	[33]
HnRNPs H1/H2	TYMP	5′-deoxyfluorouridine	Histiocytic lymphoma cell	Splicing	Resistance ↑	[34]
MTDH	Several targets	Mitomycin C, BIBF1120	Endometrial cancer cell line	Stress granule formation?	Resistance ↑	[38]

**Table 2:** miRNAs involved in the acquisition of drug resistance.

Regulators	Target genes	Anti-cancer drugs	Cell or tissue types	Effect	References
miR-93	PTEN/Akt	Cisplatin	Ovarian cancer	Resistance ↑	[43]
miR-155	Apaf-1	Cisplatin	Lung cancer	Sensitivity ↓	[44]
miR-155	PPP2CA	Cisplatin	Colon cancer	Resistance ↑	[45]
miR-135a/b	MCL1	Cisplatin	Lung cancer	Resistance ↓	[46]
miR-200c	Bmi-1	Cisplatin	Melanoma	Resistance ↓	[47]
miR-203	SOCS3	Cisplatin	Breast cancer	Sensitivity ↓	[48]
miR-181b	Bcl2	Cisplatin	Lung cancer	Resistance ↓	[49]
miR-19b	SFPQ, MYBL2	5-fluouracil	Colorectal cancer	Resistance ↑	[51]
miR-192/215	TYMS	5-fluouracil	Colorectal cancer	Resistance ↓	[52]
miR195	Bcl-w	5-fluouracil	Hepatocellular carcinoma	Resistance ↓	[53]
miR-21	hMSH6, hMSH2	5-fluouracil	Colorectal cancer	Resistance ↑	[55]
miR-20a	BNIP	5-fluouracil	Colorectal cancer	Resistance ↑	[56]
miR-17	BIM	Paclitaxel	Ovarian cancer	Sensitivity ↓	[57]
miR-31	MET	Paclitaxel	Ovarian cancer	Resistance ↓	[58]
miR-34c	Bmf	Paclitaxel	Lung cancer	Resistance ↑	[59]
miR-451	14-3-3ζ	Tamoxifen	Breast cancer	Resistance ↓	[63]
miR-221/222	p27	Tamoxifen	Breast cancer	Resistance ↑	[64]
miR-21	PDCD4	Arsenic trioxide	Leukemia	Resistance ↑	[65]
miR-708	EYA3	Etoposide, Doxorubicin	Ewing sarcoma	Resistance ↓	[66]
miR-211	MMP9	Temozolomide	Glioma	Sensitivity ↑	[67]
miR-215	DTL, DHFR, TYMS	Methotrexate, Tomudex	Osteosarcoma, Colorectal cancer	Sensitivity ↓	[68]
miR-143	IGF-IR	Oxaliplatin	Colorectal cancer	Sensitivity ↑	[69]
